# Where India Stands in Malaria Elimination?

**DOI:** 10.3389/fpubh.2014.00098

**Published:** 2014-07-28

**Authors:** Sunil Dhiman, Pronobesh Chattopadhyay

**Affiliations:** ^1^Defence Research Laboratory, Defence R&D Organisation, Tezpur, India

**Keywords:** malaria, insecticide, insecticide-treated bed nets, WHO, elimination

Malaria elimination campaign during the end of the last century relied on insecticides and malaria treatment, but this effort did not succeed in many endemic countries and collapsed after a few decades. India is among the 35 countries, which contribute 96% of total malaria cases and 98% of total malaria deaths in the world (1). Recent studies suggest that the neighboring countries of India are at the edge of eliminating malaria. In Sri lanka, the key component to achieve malaria cases reduction by 99.9% was the ability to be flexible and adapt to changing conditions (2, 3). Mobile malaria clinics were deployed to protect hard-to-reach and displaced population groups. Long-lasting insecticide-treated nets were distributed by engaging non-governmental organizations local to the endemic areas. Bhutan has initiated the use of mobile phones to collect and map information on the location of households, breeding sites, malaria cases, and settlements (4, 5).

In India, the malaria control program was initiated in 1953 with an aim of eradication; however, still a number of outbreaks are occurring in different parts of the country. Recent malaria report published by National Vector Borne Diseases Control Program (NVBDCP), Ministry of Health and Family Welfare, and the government of India revealed that there were about 0.88 million confirmed malaria cases and 440 deaths attributed to malaria in 2013 (6). The overall malaria cases and deaths have decreased in recent years in India, but a study funded by NIH, USA and published in Lancet argues that more than 0.2 million Indians die of malaria annually (7). The study further reports that about 90% of malaria related deaths were in rural areas while about 86% took place away from the health centers. A recent study has suggested that there is a certain population in India that does not have access to medical facilities for one or another reason, which further strengthen the claim that many deaths might occur at home without reaching to any reporting facility (8). The actual estimate of malaria mortality in India has been argued in recent years (8, 9). If the data reported in Lancet happen to be true, the substantial underreporting of malaria cases could prove to be a serious disruption of WHO’s anti-malaria effort, which estimated that India is moving toward the “pre-elimination phase” by 2015.

## Conflict of Interest Statement

The authors declare that the research was conducted in the absence of any commercial or financial relationships that could be construed as a potential conflict of interest.
